# Effect of Alkali Metal Atoms Doping on Structural and Nonlinear Optical Properties of the Gold-Germanium Bimetallic Clusters

**DOI:** 10.3390/nano7070184

**Published:** 2017-07-17

**Authors:** Xiaojun Li, Shuna Li, Hongjiang Ren, Juxiang Yang, Yongqiang Tang

**Affiliations:** The Key Laboratory for Surface Engineering and Remanufacturing in Shaanxi Province, School of Chemical Engineering, Xi’an University, Xi’an 710065, China; lishuna101@163.com (S.L.); hjren@xawl.edu.cn (H.R.); juxiangyang03@126.com (J.Y.); yqtang11@163.com (Y.T.)

**Keywords:** semiconductor clusters, electronic structures, chemical bondings, spherical aromaticity, NLO properties

## Abstract

A new series of alkali-based complexes, AM@Ge*_n_*Au (AM = Li, Na, and K), have been theoretically designed and investigated by means of the density functional theory calculations. The geometric structures and electronic properties of the species are systematically analyzed. The adsorption of alkali metals maintains the structural framework of the gold-germanium bimetallic clusters, and the alkali metals prefer energetically to be attached on clusters’ surfaces or edges. The high chemical stability of Li@Ge_12_Au is revealed by the spherical aromaticity, the hybridization between the Ge atoms and Au-4*d* states, and delocalized multi-center bonds, as well as large binding energies. The static first hyperpolarizability (*β*_tot_) is related to the cluster size and geometric structure, and the AM@Ge*_n_*Au (AM = Na and K) clusters exhibit the much larger *β*_tot_ values up to 13050 a.u., which are considerable to establish their strong nonlinear optical (NLO) behaviors. We hope that this study will promote further application of alkali metals-adsorbed germanium-based semiconductor materials, serving for the design of remarkable and tunable NLO materials.

## 1. Introduction

Alkalis metals have attracted much attention because they serve as building blocks for novel materials with tunable properties [1], and a great deal of new alkali-based complexes have been recently reported [2,3,4]. In particular, a series of alkalides, proposed as the candidates of nonlinear optical (NLO) materials, exhibit exceptionally large NLO responses and are promising for their spectacular semiconductor potentials in photoelectricity devices and optical communications, e.g., M@C_60_ (M = Li, Na, Cs) [5], Li@C_60_-BX_4_ (X = F, Cl, Br) [6], Li*_n_*F (*n* = 2–5) [7], Li_2_@BN nanotubes [8], OLi_3_-M-Li_3_O (M = Li, Na, K) [9], Li_3_^+^(calyx[4]pyrrole)M^−^ [10], Li(NH_3_)_4_M (M = Li, Na, K) [11], and Li(CH_3_NH_2_)*_n_*Na [12], etc., in which both theoretical and experimental studies play a crucial role in finding ways to enhance the NLO behaviors. On the other hand, the different NLO materials can be expressed by the largely high second-order NLO response at the molecular level [13,14], and the first hyperpolarizability can be quantitatively used to evaluate the potential NLO materials of alkali-based complexes. 

Meanwhile, owing to the unique electronic properties of alkalides, their derivatives have been extensively reported with the extension to silicon-based clusters, e.g., Si_10_(Li, Na, K)*_n_* (*n* = 1, 2) [15], (Li, Na, K)@Si*_n_*Nb (*n* = 1–12) [16], Si_10_Li_8_ [17], and Si*_n_*Fe (*n* = 1–14) [18], which ascribe to loosely bound electrons in alkali-based complexes, resulting in the large NLO responses. It is also noteworthy that although the analogous germanium and silicon species are isovalent, their structures and physicochemical properties are quite different [19,20,21,22,23,24,25]. For example, the static hyperpolarizabilities of pure Si*_m_*Ge*_n_* (*m* + *n* = 7, *n* = 0–7) clusters were studied by using the density functional theory (DFT) and MP2 ab initio methods [26], and revealed that the enhancement of the hyperpolarizabilities arises from more polarizable character on the germanium atoms, and thus alkali metals-adsorbed germanium-based semiconductor clusters may have the unusual features in high-performance optoelectronic devices for the potential applications. In addition, Knoppe and Ozga et al. [27,28,29] found that the gold-containing clusters appear to have strong NLO responses, and the Au atom plays an important role in the optoelectronic application. Obviously, the knowledge of geometries, electronic structures, chemical bonding, and nonlinear optical properties of the species is very important for understanding these applications, but these are rarely reported. With this motivation, we explored the stability and electronic structures of alkali metals-adsorbed Ge*_n_*Au semiconductor clusters for the first time, labeled as AM@Ge*_n_*Au (AM = Li, Na, and K; *n* = 2–13), and investigated the effect of alkali metals on the dipole moment, polarizability, and first hyperpolarizability (*β*_tot_). The results suggest that the AM@Ge*_n_*Au (AM = Na and K) clusters may be proposed as novel potential high-performance NLO materials, especially for the Na@Ge_7_Au cluster with a large *β*_tot_ value.

## 2. Computational Details

The geometrical optimizations of the AM@Ge*_n_*Au (AM = Li, Na, and K; *n* = 2–13) clusters were carried out by using the hybrid DFT-B3LYP functional [30,31], implemented in the Gaussian 09 suite of programs [32]. This method provides a good prediction on energy evaluation [33,34,35], in conjunction with the Karlsruhe split-valence basis set augmented with polarization functions (def-SVP) for all alkali metal atoms and the double-ζ LanL2DZ [36,37,38] with effective core potentials (ECPs) for the Ge and Au atoms, and then the low-lying isomers are further reoptimized at the B3LYP/def-TZVP level of theory. In the calculations, the singlet and triplet spin states were examined for each initial structure, and the zero-point vibrational correction was included into the relative energies. According to the previous works on the neutral [24] and cationic [39] Ge*_n_*Au clusters, we searched a large number of initial isomers for the alkali metals-adsorbed gold-doped germanium clusters from the following steps. One is that the alkali metal atoms are attached to different germanium positions on the surface, edge or apex of the lowest-energy structures of the Ge*_n_*Au clusters. The second is the attaching structure with gold positions adsorbed by alkali metals. The remaining structures were constructed by us. Vibrational frequency computations were conducted to ensure that these low-lying structures are local minima on its potential energy surface.

The energy of a molecular system in the presence of a homogeneous electric field can be written as the following equation [40,41,42]:

(1) $$E(F)=E^{0}-\mu_{i}F_{i}-\frac{1}{2}\alpha_{ij}F_{i}F_{j}-\frac{1}{6}\beta_{ijk}F_{i}F_{j}F_{k}-\frac{1}{24}\gamma_{ijkl}F_{i}F_{j}F_{k}F_{l}\cdot \cdot \cdot$$

Here, *E*^0^ is the total energy of the molecule without electric field present, *F_i_* is the electric field component in the *α* direction; the *μ_i_*, *α_ij_*, *β_ijk_* terms are the dipole, the polarizability, and the first hyperpolarizability, respectively. The static dipole moment (*μ*_0_) and polarizability (*α*_iso_) are defined as follows:

*μ*_0_ = (*μ*_x_^2^ + *μ*_y_^2^ + *μ*_z_^2^)^1/2^ (2)

(3) $$\alpha_{\mathrm{iso}}=(\alpha_{xx}+\alpha_{yy}+\alpha_{zz})/3$$

The static first hyperpolarizability (*β*_tot_) is obtained as follows:

*β*_tot_ = (*β*_x_^2^ + *β*_y_^2^ + *β*_z_^2^)^1/2^ (4)

where $\beta_{i}=(1/3)\sum_{j}\left(\beta_{ijj}+\beta_{jji}+\beta_{jij}\right)$ i,j={x,y,z}.

The density-of-states (DOS) spectra were convoluted utilizing the GaussSum 2.2 program [43] with the full-width at half maximum (FWHM) of 0.3 eV. Chemical bonding analyses were performed using the adaptive natural density partitioning (AdNDP) method proposed by Zubarev and Boldyrev [44]. The molecular orbitals were plotted with the isodensity surfaces (0.02 e^1/2^/(bohr)^3/2^), and the molecular graphs were visualized using the VMD program [45]. The long-range corrected functional CAM-B3LYP [46] was utilized to calculate the excited energies within the framework of the time-dependent density functional theory (TDDFT) [47], as well as for the evaluation of linear and nonlinear (L&NLO) optical properties, e.g., dipole moments (*μ*_0_), isotropic polarizability (*α*_iso_), and first hyperpolarizability (*β*_tot_) of the AM@Ge*_n_*Au clusters.

## 3. Results and Discussion

### 3.1. Geometric Structures

**Figure 1:**
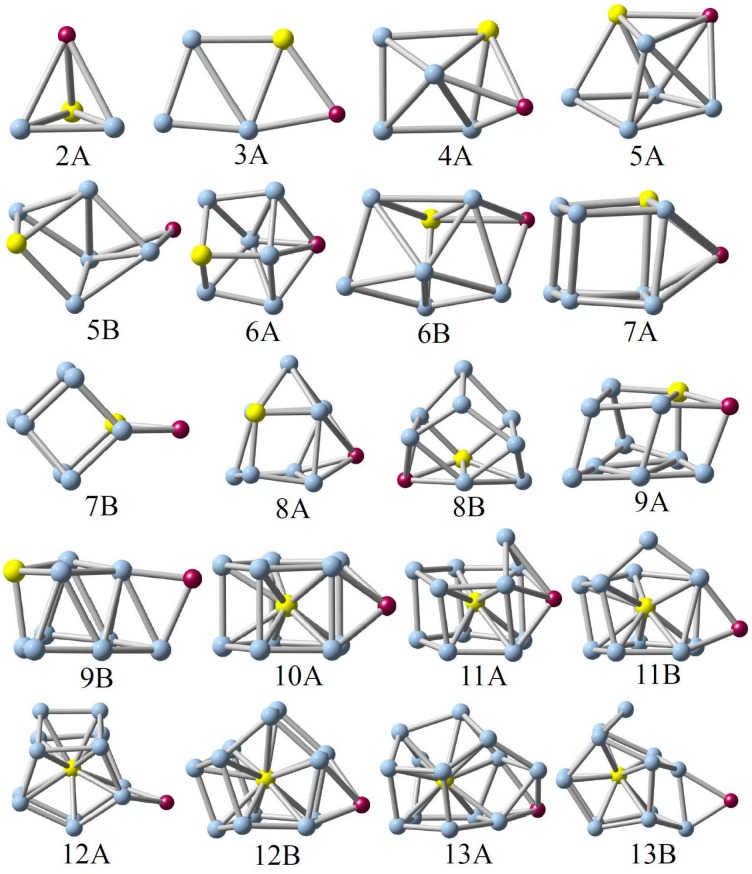
Low-lying structures of the AM@Ge*_n_*Au (*n* = 2–13) clusters, obtained by using the hybrid B3LYP functional. The blue, yellow and purple balls represent the Ge, Au and alkali metal (AM) atoms, respectively.

The low-lying structures of the AM@Ge*_n_*Au (*n* = 2–13) clusters are displayed in Figure 1. The relative energies of these low-lying isomers, obtained by using the two basis sets as mentioned above, are listed in Table S1 of Supplementary Materials, in which all the calculations indicate that the global minimum for each cluster is in singlet spin states. Meanwhile, one can see from Figure 1 that adsorption of the AM atoms does not largely change the structural framework of the Au-doped germanium clusters, as reported previously by Li et al. [24], and the alkali metals prefer to be attached on clusters’ surface or edge with the multi-adsorbed bonds (multi-bonds) rather than the AM–Ge or AM–Au single-adsorbed bond in the stable structures.

**(Li, Na, K)@Ge_2_Au**. The lowest-energy structure (2**A**) of AM@Ge_2_Au (AM = Li, Na, and K) takes the tetrahedron structure with *C_s_* symmetry, generated from the AM atoms being capped on the clusters’ surface of the triangular Ge–Ge–Au base. The corresponding triplet states are less stable in energy than those with singlet spin states by 1.03–1.43 eV high at the B3LYP/def-TZVP level of theory (see Table S1 of Supplementary Materials). According to our calculations, the equilibrium Ge–Ge bond lengths are predicted to be 2.399–2.601 Å in the AM@Ge_2_Au clusters.

**(Li, Na, K)@Ge_3_Au**. 3**A** is a planar structure with *C_s_* symmetry, which can be regarded as the alkali metals being bonded on the edge (Au–Ge) of the rhombic Ge_3_Au cluster, whereas the structure adsorbed on another edge (Ge–Ge) by alkali metals can be found to be unstable. The 3**A** geometry can be considered as the global minimum of the AM@Ge_3_Au (AM = Li, Na, and K) cluster. In Li@Ge_3_Au, the equilibrium bond lengths are evaluated to be 2.597 Å for the Li–Au bond, 2.515 Å for the Li–Ge bond, 2.558–2.722 Å for two Au-Ge bonds, and 2.353–2.876 Å for three Ge–Ge bonds.

**(Li, Na, K)@Ge_4_Au**. As previously reported [24], the ground-state structure of the Ge_4_Au cluster is a pyramid-distorted isomer. When the AM atoms are attached on the side face (Ge–Ge–Au) of Ge_4_Au, the most stable 4**A** structure is formed, in which the equilibrium Li–Ge and Li–Au bond lengths are predicted to be 2.597, 2.615, and 2.771 Å at the B3LYP/def-TZVP level of theory, respectively.

**(Li, Na, K)@Ge_5_Au**. In the search for the low-lying configurations of AM@Ge_5_Au (AM = Li, Na, and K), there are two stable structures (5**A** and 5**B**) with close energies (0.13–0.16 eV in singlet spin states) to be obtained while their triplet spin states have the higher relative energies (at least 0.78 eV) at the B3LYP/def-TZVP level of theory. The geometric differences between the two isomers are that the AM atoms are adsorbed on the side face (Ge–Ge–Ge–Au) or side edge (Ge–Ge) of the distorted triangular prism. Obviously, the global minimum of AM@Ge_5_Au (AM = Li, Na, and K) is found to be the 5**A** structure. It is noteworthily that for small clusters with *n* ≤ 5, the lowest-energy structures take the AM-adsorbed on Au-adjacent edge or surface of cluster base, to form the stable AM–Au chemical bonds.

**(Li, Na, K)@Ge_6_Au**. Similar to the *n* = 5 cluster as discussed above, we have manually designed a great number of initial geometries for the *n* = 6 cluster size, e.g., adsorbing the AM atoms to different position sites and substituting the apical Ge atom by the AM atoms in the Ge_7_Au cluster, and so on, but the 6**A** cagelike with *C_s_* symmetry is the lowest-energy structure for the AM@Ge_6_Au (AM = Li, Na, and K). The 6**B** isomer is a distorted quadrangular prism, which is less stable than 6**A** by at least 0.13 eV higher in energy. Meanwhile, it is found that the two different basis sets used herein provide the same energetic ordering for these small clusters (see Table S1 of Supplementary Materials).

**(Li, Na, K)@Ge_7_Au**. As mentioned above, the AM atoms prefer energetically to be adsorbed on the Au-adjacent surface (Ge–Ge–Ge–Au) or edge (Ge–Au) of the quadrangular prism (QP), thus the two stable structures (7**A** and 7**B**) can be obtained. According to our calculation, the 7**A** isomer is predicted as a putative global minimum for the (Li, K)@Ge_7_Au clusters, whereas the 7**B** isomer is the most stable structure for Na@Ge_7_Au.

**(Li, Na, K)@Ge_8_Au**. The 8**A** and 8**B** isomers are one bicapped square prism, which can be viewed as the alkali metal and Au atom capped on different faces of the lowest-energy Ge_7_Au cluster (called as square prism). Of which 8**A** is the lowest-energy structure for the Li@Ge_8_Au cluster at the B3LYP/LanL2DZ(Ge,Au)/def-SVP(AM) level of theory, while the most stable structure for (Na, K)@Ge_8_Au is the 8**B** isomer lying only 0.02–0.04 eV under the 8**A** isomer. However, the energetic ordering of 8**A** and 8**B** for (Na, K)@Ge_8_Au can be reversed at the B3LYP/Def-TZVP level of theory. Thus, the 8**A** geometry can be found to be the global minimum for the AM@Ge_8_Au (AM = Li, Na, and K) cluster at the B3LYP/Def-TZVP level of theory.

**(Li, Na, K)@Ge_9_Au**. Based on the lowest-energy Ge_9_Au cluster [24], the alkali metal-adsorbed complexes can be generated from the AM atoms attaching on the above Au-Ge layer. It is obvious that the Au-adjacent isomer (9**A**) is more stable than the Au-outlying one (9**B**) by ~0.04–0.06 eV in singlet spin states, being consistent with the small clusters (*n* ≤ 5).

**(Li, Na, K)@Ge_10_Au**. The Ge_10_Au cluster is a *C*_2*v*_-symmetrical pentagonal prism with the Au-encapsulated into the central position of structure [24]. When the alkali metal atoms are directly face-capped on side quadrangle of Ge_10_Au, a new equilibrium 10**A** structure, considered as global minimum, is yielded using the DFT-B3LYP functional. Of which the theoretical bond lengths are predicted to be 2.769 Å for the four equivalent Li−Ge bonds, and 2.715, 2.733, and 2.742 Å for three kinds of the equivalent Ge–Au bonds.

**(Li, Na, K)@Ge_11_Au**. The 11**A** structure can be regarded as the Ge atom being directly capped on the side edge of the above pentagon of 10**A** or alkali metal atoms being directly adsorbed on the face-sided pentagon of the lowest-energy Ge_11_Au cluster, as published previously [24]. Similarly, the low-lying 11**B** isomer can be formed by capping the alkali metal atoms on the face-sided quadrangle of the Ge_11_Au cluster, which is less stable than 11**A** by at least 0.07 eV high in energy (see Table S1 of Supplementary Materials ).

**(Li, Na, K)@Ge_12_Au**. Similar to the *n* = 11 cluster as discussed above, the 12**A** and 12**B** structures can be generated from the different position sites adsorbed by alkali metal atoms on the lowest-energy Ge_12_Au cluster. Interestingly, the 12**B** isomer has a highly coordinated surface adsorbed by the Li atom to eliminate the number of dangling bonds on the clusters’ surface, regarded as the global minimum of Li@Ge_12_Au. Contrastingly, the adsorption of Na/K in lower coordinated edge position (12**A**) will form the lowest-energy structure for (Na, K)@Ge_12_Au cluster.

**(Li, Na, K)@Ge_13_Au**. Referring to the equilibrium geometries of the low-lying Ge_13_Au cluster reported previously [24], a great number of initial structures of alkali metal-adsorbed complexes were extensively constructed and optimized in order to locate the global minimum of the cluster size. For comparison, we only show two first stable 13**A** and 13**B** isomers for (Li, Na, K)@Ge_13_Au (see Figure 1), which shows the close energy difference between 0.03 eV and 0.22 eV at the B3LYP/Def-TZVP level of theory (see Table S1 of Supplementary Materials). Obviously, the lowest-energy 13**A** structure undergoes the structural relaxation, whereas the low-lying 13**B** structure keeps the structural framework of the lowest-energy Ge_13_Au cluster.

### 3.2. Chemical Stability and Electronic Structures

**Figure 2:**
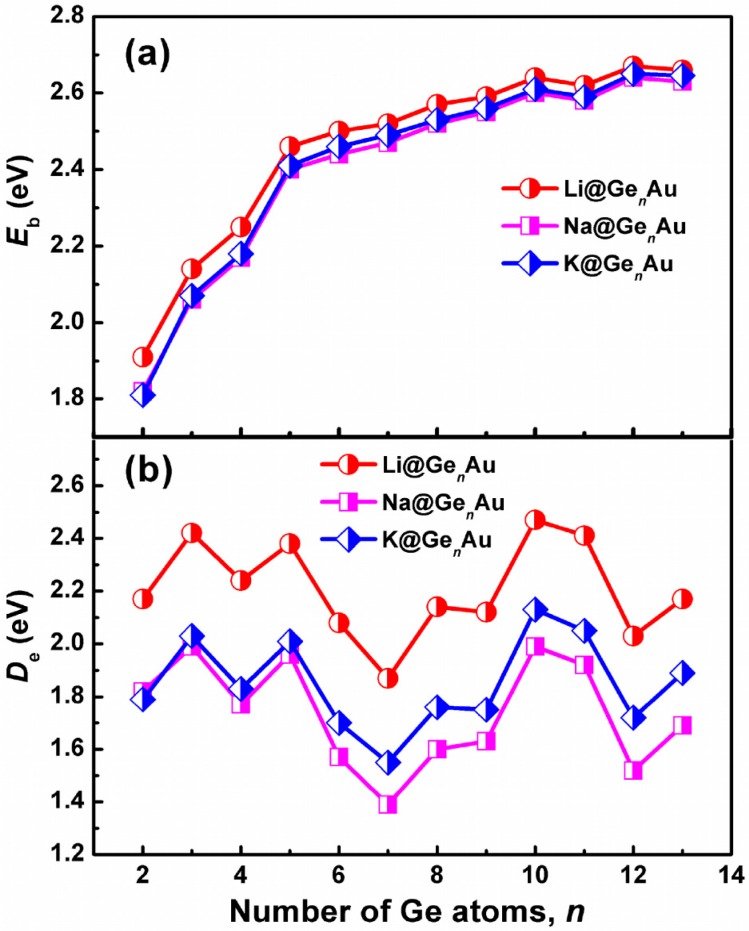
Size dependences of (**a**) the average binding energies (*E*_b_) and (**b**) dissociation energies (*D*_e_) for the AM@Ge*_n_*Au (*n* = 2–13) clusters, obtained by using the hybrid B3LYP functional, in conjunction with the Def-TZVP basis set.

**Figure 3:**
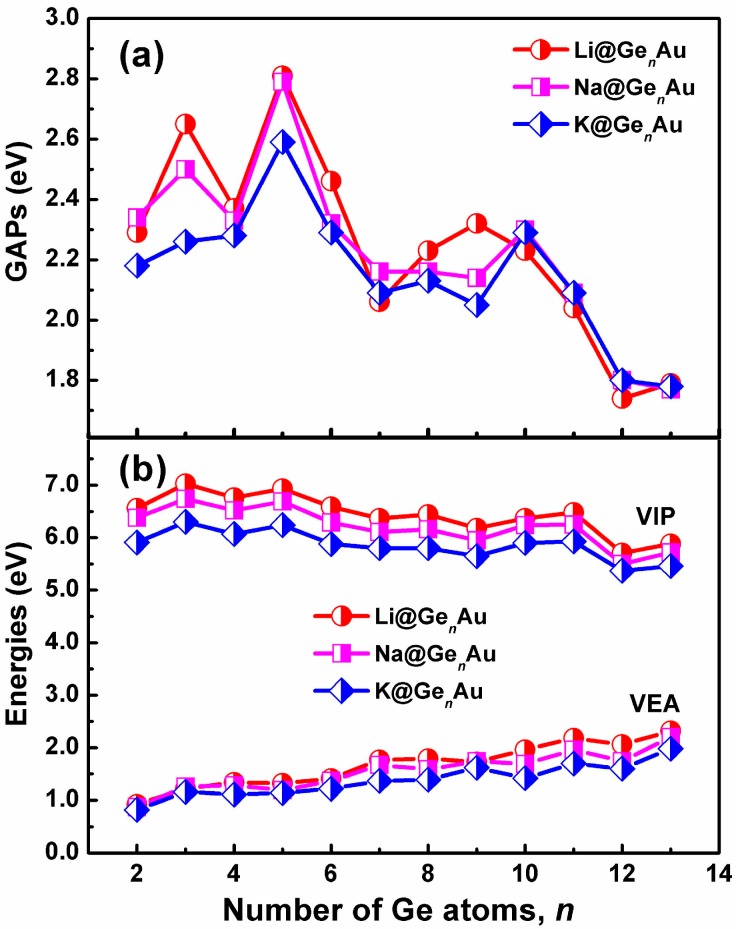
Size dependences of (**a**) HOMO-LUMO gaps (GAPs), and (**b**) vertical ionization potentials (VIP) and vertical electron affinities (VEA) for the AM@Ge*_n_*Au (*n* = 2–13) clusters, obtained by using the hybrid B3LYP functional, in conjunction with the Def-TZVP basis set.

In order to explore the size selectivity and the electronic properties of the AM@Ge*_n_*Au (*n* = 2–13) clusters, we plotted the average binding energy (*E*_b_), the dissociation energy (*D*_e_, AM@Ge*_n_*Au → Ge*_n_*Au + AM), the HOMO-LUMO gaps (GAPs), and the vertical ionization potentials (VIP) and vertical electron affinities (VEA) in Figure 2 and Figure 3, respectively, which are defined by:

*E*_b_(*n*) = [*E*(AM) + *nE*(Ge) + *E*(Au) − *E*(AM@Ge*_n_*Au)]/(*n* + 2) (5)

*D*_e_(*n*) = *E*(Ge*_n_*Au) + *E*(AM) − *E*(AM@Ge*_n_*Au) (6)

where the *E* term represents the total energy with zero-point vibrational corrections.

It is well known that a larger *E*_b_ value indicates a higher chemical stability of cluster. We can see from Figure 2a that the average binding energies of AM@Ge*_n_*Au (AM = Na and K) have almost similar values for each cluster size, which are slightly lower than that of Li@Ge*_n_*Au by ~0.03–0.10 eV. However, all of the AM@Ge*_n_*Au (AM = Li, Na, and K) clusters show the same increased trends. For example, their average binding energies dramatically increase up to the size of *n* = 5 and smoothly increase with the size *n* = 6–13, indicating that the large-sized doped clusters are more stable than the small-sized ones, especially for the *n* = 10 and 12 clusters. The dissociation energy (*D*_e_) with the removal of the AM atoms is another useful physical quantity that can also reflect the relative stability of AM@Ge*_n_*Au. It is apparent from Figure 2b that the *n* = 3, 5, and 10 clusters are the local maximum *D*_e_ peaks, which are more stable than their corresponding neighbors. Meanwhile, the *D*_e_ energetic ordering by alkali metals is Li > K > Na.

The HOMO-LUMO gaps (GAPs) of the AM@Ge*_n_*Au clusters are listed in Figure 3a. One can find that the gaps (2.04–2.81 eV) in the small-sized clusters with the size *n* ≤ 11 are larger than those, 1.74–1.80 eV, in the large-sized clusters. Clearly, the HOMO-LUMO gap values with *n* = 12 and 13 lie in the typically optical region (e.g., less than 2 eV), making these clusters attractive for the cluster-assembled optoelectronic materials, which are consistent with previous reports[48], e.g., Zn@Ge_12_ and Cd@Sn_12_.

The vertical ionization potentials (VIPs) and vertical electron affinities (VEAs) of AM@Ge*_n_*Au (*n* = 2–13) are considered to explore the dependence of electronic structures on the cluster size. The VIP can be evaluated by the energy difference between the optimized neutral species and single-point cationic species at the optimized neutral geometry. The VEA can be computed by adding one electron to the neutral species in its equilibrium geometry and taking their energy differences. The calculated VIPs and VEAs of the most stable clusters are plotted in Figure 3b. As shown in Figure 3b, the curve reveals that there is a gradually decreased behavior for VIPs along with increasing number of Ge atom, and the cluster with small VIPs (e.g., *n* = 12) will be more close to a metallic species [49]; reversely, there is a gradually increased trend for VEAs. Similar to the *E*_b_ and *D*_e_ values, the Li@Ge*_n_*Au clusters give the larger VIPs and VEAs than the Na- and K-based complexes. It is notable that the VIPs of AM@Ge_10_Au (AM = Na and K) are 5.48 eV and 5.32 eV, respectively, smaller than that of Li (5.62 eV) and Na (5.40 eV), respectively, indicating these clusters should have an electronic structure reminiscent of an alkali atom.

### 3.3. Density of States

**Figure 4:**
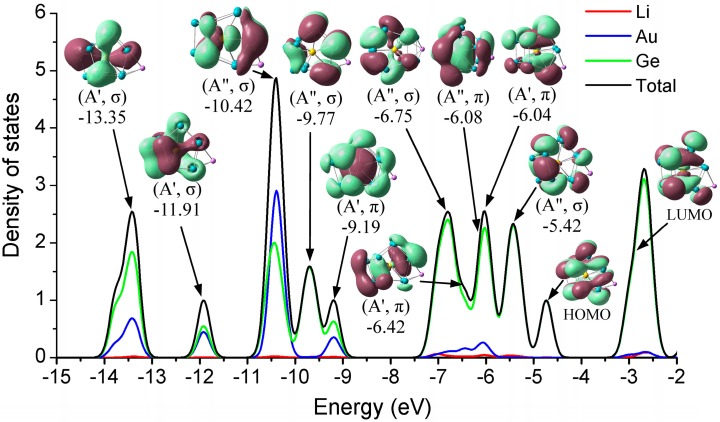
Total (DOS) and partial (PDOS) density of states of the most stable Li@Ge_12_Au (12**B**) cluster, and the valence orbitals are obtained at the B3LYP/def-TZVP level of theory. The atomic contributions (Li, Au, and Ge) of the cluster to the DOS spectrum are labeled.

To further investigate the electronic effect of the alkali metals adsorption on the clusters’ surface, we explored the total (TDOS) and partial (PDOS) density of states, taking the stable Li@Ge_12_Au (12**B**) cluster as a typical case, shown in Figure 4, which includes all atomic contributions (Li, Ge, and Au) of the clusters to PDOS, while the contributions of different atomic shells (*s*, *p*, *d*) to the molecular orbitals (MOs) are also discussed.

As for this case, each Ge atom is expected to contribute its four valence electrons (4*s*^2^4*p*^2^) to the shell configuration while the Li and Au atoms can contribute one (2*s*^1^) and eleven (5*d*^10^6*s*^1^) valence electrons, respectively. Thus, the Li@Ge_12_Au cluster with *C*_s_ symmetry contains 60 valence electrons, which are distributed in the following orbital configuration: (1a′)^2^(2a′)^2^(1a″)^2^(3a′)^2^(2a″)^2^ (3a″)^2^(4a′)^2^(5a′)^2^(6a′)^2^(4a″)^2^(5a″)^2^(7a′)^2^(8a′)^2^(6a″)^2^(9a′)^2^(7a″)^2^(10a′)^2^(11a′)^2^(12a′)^2^(13a′)^2^(8a″)^2^(14a′)^2^(15a′)^2^ (9a″)^2^(16a′)^2^(10a″)^2^(17a′)^2^(11a″)^2^(18a′)^2^(12a″)^2^. The main valence molecular orbitals (0.02 e/a.u.^3^) of the cluster are depicted in Figure 4. We can clearly see that, for the stable Li@Ge_12_Au (12**B**) clusters, the *σ*-type HOMO has an interesting double pumpkin shape with the electron cloud delocalized on the two sides of the Ge–Ge–Au cross section, mostly involving the five-membered Ge_5_ rings, due to the hybridization of 12.38% Ge-4*s* and 85.51% Ge-4*p* states, whereas the *σ*-type LUMO originates mainly from the dominant interactions between Au-5*d*_xz_ state and 4*s*4*p* hybridized orbitals of the Ge_12_ cage. The Au-5*d*_xz_ orbital has only 3.37% contribution to the LUMO, while the 4*s*4*p* hybridized orbitals of 12 Ge atoms have 93.70% contribution to the LUMO. Obviously, the small HOMO-LUMO gap (1.74 eV) of the cluster should be largely related to the electron distributions of the frontier molecular orbitals, and the hybridization of Ge atoms with metal dopants can increase the HOMO-LUMO gap, and enhance the chemical stability of the cluster [22]. The strongest DOS band at around −10.42 eV mainly ascribes to the *σ*-type HOMO-19 orbital, which comes mostly from different atomic shells, e.g., 66.77% Au-*d*_yz_, 23.19% Ge-4*s*, and 2.92% Ge-4*p*_y_ states. In addition, the Au-4*d* states have strong interactions (28.86%, 46.57%, and 14.26%) with Ge atoms for HOMO-22 (−13.35 eV), HOMO-21 (−11.91 eV), and HOMO-12 (−9.19 eV) orbitals, respectively, indicating that the 4*d* states of Au atom in these orbitals are involved in the chemical bonding. One can see that the valence electron orbitals of Li@Ge_12_Au are divided into two different subsets occupied by σ and π electrons, and the doped cluster contains eight valence π-electrons in four molecular orbitals, e.g., −9.19(A′, π), −6.42(A′, π), −6.08(A″, π), and −6.04(A′, π) orbitals, which belong to one 1S- and three 1P-subshells of the doped cluster, according to the electron shell model [22,50,51].

### 3.4. Chemical Bonding Analysis

In order to further explore the bonding properties of metal dopant and Ge atoms, we performed the adaptive natural density partitioning (AdNDP) analysis proposed by Zubarev and Boldyrev [44], taking the most stable Li@Ge_12_Au (12**B**) cluster as an example. The AdNDP method is based on the concept of electron pairs as the main elements of chemical bonding, and represents the electronic structure in terms of *n*-center two-electron (*n*c-2e) bonds, in which the *n* values go from one (lone-pair) to the maximum number of clusters. This method has been successfully applied to gain insight into the bonding characters not only for fullerene derivatives [41], but also for boron [52,53,54,55] and transition-metal doped Si/Ge clusters [22].

**Figure 5:**
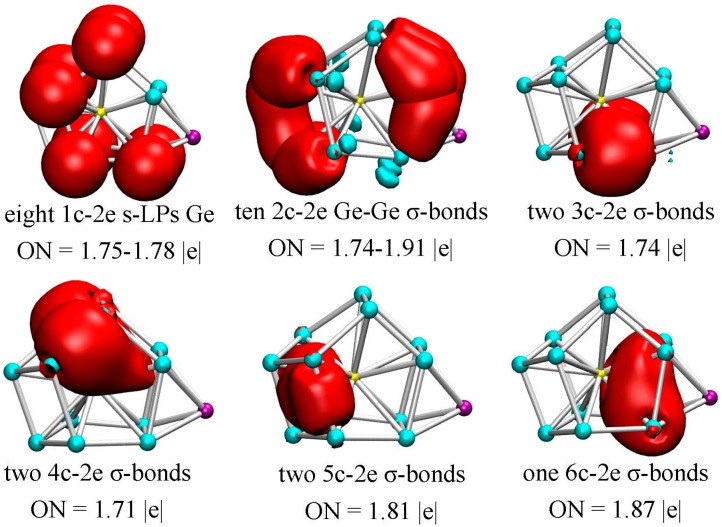
AdNDP chemical bonding analyses of the Li@Ge_12_Au (12**B**) cluster. ON denotes the electron occupation number and is close to the ideal population of 2.00 |e|.

In the method, the occupation numbers (ONs) indicate the number of electrons per bond, and the ONs exceed the established threshold value and are close to the ideal limit of 2.00 |e|. As mentioned above, the Li@Ge_12_Au (12**B**) cluster has 60 valence electrons and thus 30 chemical bonds with each bond occupied by two electrons. According to the AdNDP results, there are five typical *d*-lone pairs (LPs) on Au with ON = 1.93–1.98 |e|, and eight *s*-lone pairs on the Ge atoms with ON = 1.75–1.78 |e| (see Figure 5), which reveals that electrons on the Ge atoms are not completely localized into lone pairs, but partially participate into the localized or delocalized bondings. As can be seen from Figure 5, ten two-center two-electron (2c-2e) σ-bonds are localized on clusters’ surface with ON = 1.74–1.91 |e|. Meanwhile, a total of seven delocalized σ-bonds can be readily identified: two 3c-2e σ-bonds (ON = 1.74 |e|) on the Ge-Au-Ge triangles under the cage structure, two 4c-2e σ-bonds (ON = 1.71 |e|) on the top structure, two 5c-2e σ-bonds (ON = 1.81 |e|) on the tetragonal pyramid of left structure and one 6c-2e σ-bonds (ON = 1.87 |e|) on the tetragonal bipyramid of right structure with two vertices occupied by Au and Li atoms. Obviously, the localized 2c-2e σ-bonds are mainly located on the clusters’ surface, whereas all of the delocalized σ-bonds are always involved in the endohedral Au dopant. This indicates that the delocalized Au–Ge interactions will be responsible for the structural stabilization of the lowest-energy Li@Ge_12_Au (12**B**) cluster.

### 3.5. Spherical Aromaticity

It is well known that aromaticity is one of important measures of many compounds, and analogous aromatic compounds commonly take on high chemical stability relative to non-aromatic ones. For planar structures, the aromatic characters are identified by using the 4*N* + 2 Hückel rule [56]. However, In 2000, Hirsch et al. [57] proposed another electron counting rule for three-dimensional (3D) structures, namely 2(*N* + 1)^2^ rule, which has been proven as an effective aromaticity criterion, with the extension to inorganic clusters [22,50,58]. In the proposal, the π-electron system of the spherical species can be approximately regarded as a spherical electron gas, which surrounds the spherical surface [59]. According to the Pauli principle, if the number of π electrons in a spherical species satisfies the 2(*N* + 1)^2^ counting rule, and then the 3D structures can be considered to be aromatic. 

Figure 4 shows the molecular orbitals of the stable Li@Ge_12_Au (12**B**) cluster. One can note that the valence electron orbitals are divided into two different orbital sets occupied by σ or π electrons. In particular, the Li@Ge_12_Au (12**B**) cluster contains eight π-electrons in four molecular orbitals as mentioned above, and these π-electrons fully satisfy the 2(*N*_π_ + 1)^2^[*N*_π_ = 1] counting rule. As a result, the π-electron species makes Li@Ge_12_Au spherically aromatic, and the aromatic character can be regarded as one of the main reasons in the structural stabilization of endohedrally cluster. However, the 2(*N* + 1)^2^ electron counting rule cannot be solely applied to explore the aromaticity of compounds, e.g., the bianionic Si_12_^2−^ cluster contains eight π-electrons, but is predicted to be antiaromatic [60]. Therefore, the aromaticity of the Li@Ge_12_Au (12**B**) cluster has to be further confirmed by the nucleus-independent chemical shifts (NICS) calculations proposed by Chen and co-workers [61], on the basis of magnetic shieldings with GIAO approximation. Aromaticity is expected to be estimated by a negative NICS value, whereas antiaromaticity is expected to be estimated by a positive NICS value. In the study, a ghost atom is placed at the center of spherical geometry to compute the NICS value. At the B3LYP/def-TZVP level of theory, it is found that the NICS(0) value is −295.7 ppm for the Li@Ge_12_Au (12**B**) cluster. Thus, the aromatic character of the cluster, identified by the 2(*N*_π_ + 1)^2^[*N*_π_ = 1] counting rule, can be confirmed by the largely negative NICS values, and the large diatropic NICS(0) value can contribute to the high chemical stability of the geometry.

### 3.6. Linear and Nonlinear Optical Properties

**Figure 6:**
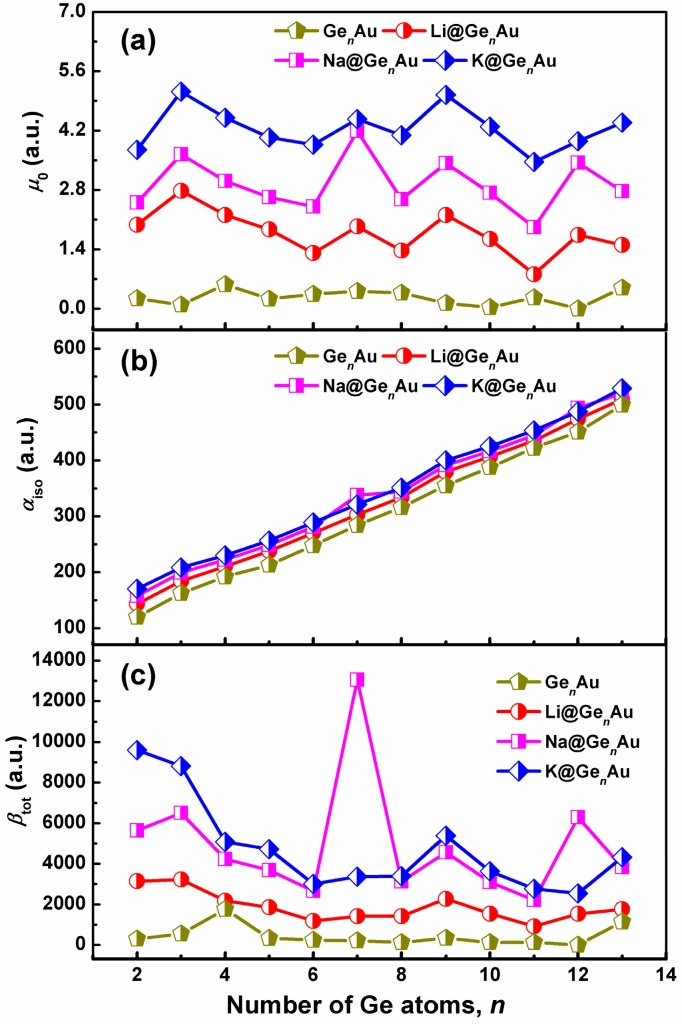
Comparison of evaluated (**a**) dipole moments (*μ*_0_), (**b**) isotropic polarizabilities (*α*_iso_), and (**c**) static first hyperpolarizability (*β*_tot_) of AM@Ge*_n_*Au (AM = Li, Na, and K; *n* = 2–13) with Ge*_n_*Au cluster, calculated at the CAM-B3LYP/def2-TZVPD level of theory.

In order to explore the L&NLO behavior of the alkali metals-adsorbed gold-doped germanium clusters, we have computed the static dipole moments (*μ*_0_), isotropic polarizabilities (*α*_iso_), and static first hyperpolarizability (*β*_tot_) using the long-range corrected CAM-B3LYP functional in conjunction with the def2-TZVPD basis sets, shown in Figure 6. According to the results, it shown in Figure 6 that the doping of alkali metals (Li, Na and K) on the clusters’ surface largely enhances the electric properties of the considered systems.

It is evident that the dipole moments of AM@Ge*_n_*Au take on the fluctuating behaviors with the increasing number of Ge atom, and the local maximum peaks are found at *n* = 3, 7, and 9 for the alkali-based complexes, which are much larger than those of bare Ge*_n_*Au clusters, see Figure 6a. However, what is different is that the isotropic polarizability of all these complexes increases linearly with increasing cluster size, as shown in Figure 6b, similar to the Li_2_-doped boron nitride clusters (*n* = 4–8) [8]. In particular, the doping of the K atom can be predicted to improve the *α*_iso_ values by ~7–41%, indicating that the alkali metal atoms provide the possibility for inducing the isotropic polarizability. Additionally, according to the hard soft acids bases (HSAB) principle [62], the species with small HOMO-LUMO gaps are less hard and more polarizable, and this reveals that the large polarizabilities of the studied species have close relations with their small energy gaps.

As shown in Figure 6c, the variation of the first hyperpolarizabilities (*β*_tot_) is interesting and has become the focus of our attention. One can see that the *β*_tot_ values of all the complexes distinctly decrease up to the size of *n* = 6, and are slightly fluctuating for larger sizes, with the exception of local maximum peaks, e.g., *n* = 7, 9, and 12. Compared with the bare Ge*_n_*Au clusters, it is found that the alkali metals can dramatically enhance *β*_tot_, but there are strong dependencies on the cluster size, and they are more sensitive to the geometric structures. As shown in Figure 6c, the ordering of the enhanced *β*_tot_ values by alkali metals is nearly K > Na > Li, with exception of the Na@Ge_7_Au and Na@Ge_12_Au clusters. Clearly, the first hyperpolarizabilities of AM@Ge*_n_*Au (AM = Na and K) are large enough to establish their strong nonlinear optical response, due to increased *β*_tot_ values (~140–6111%) induced by the two alkali metal atoms. Especially, the Na@Ge*_n_*Au (*n* = 7 and 12) and K@Ge*_n_*Au (*n* = 2 and 3) clusters possess remarkable NLO responses, with the *β*_tot_ values of 13,050, 6288, 9602, and 8812 a.u., respectively. Furthermore, the largest *β*_tot_ value (13,050 a.u.) of Na@Ge_7_Au is comparable to those of Li_2_F (12,347 a.u.) [7] and Li_2_@BN-clusters(8,0) (12,282 a.u.) [8]. On the other hand, the largest *β*_tot_ values is also about 1.87 times larger than that of the Na@Si_9_Nb^+^ cluster (6987 a.u.), which has the largest *β*_tot_ value among the AM@Si*_n_*Nb^+^ clusters reported previously [16], and it shows that the germanium-based clusters doped by alkali metals provide the greater *β*_tot_ than the silicon-based clusters. This result suggests that the germanium-based clusters with the large *β*_tot_, served as building blocks with tunable properties, may be promising for the design of novel macroscopic NLO materials. 

To further understand the NLO behavior, we have performed the TDDFT calculations on the clusters with large *β*_tot_ values at the CAM-B3LYP/def2-TZVPD level of theory. The most widely common two-level model is considered and it gains more insight into the NLO response [40,63]. The static first hyperpolarizability can be expressed by the following equation:

(7) $$\beta_{\mathrm{tot}}\propto \frac{\Delta \mu \cdot f_{0}}{\Delta E^{3}}$$

where ∆*E*, *f*_0_, and ∆*μ* are the transition energy, the oscillator strength, and the difference in dipole moment between the ground state and the crucial excited state, respectively. Of which the crucial excited state is specified by the largest *f*_0_ value, and the third-power of transition energy is inversely proportional to the *β*_tot_ value. The calculated ∆*E*, *f*_0_, and ∆*μ* values by TDDFT for the crucial excited states are listed in Table 1. One can see that the ∆*E* values for K@Ge_2_Au, K@Ge_3_Au, and Na@Ge_7_Au are 6.056 eV, 5.702 eV and 3.798 eV, respectively. The smallest ∆*E* corresponds to the first excited state of the Na@Ge_7_Au cluster, which is in accordance with its largest *β*_tot_ value. Thus, the two-level approximation can be used for qualitative description of the polarization mechanism, and the low transition energy is the most significant factor in designing the nonlinear optical materials.

**Figure 7:**
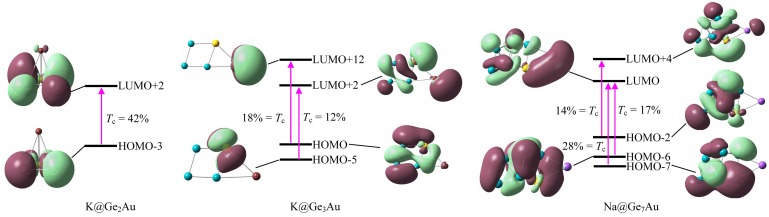
Frontier molecular orbitals involving in the crucial transitions (*T*_c_) for the K@Ge_2_Au, K@Ge_3_Au, and Na@Ge_7_Au clusters, computed at the CAM-B3LYP/def2-TZVPD level of theory.

In order to explore the origin of the second-order NLO response, the molecular orbitals (MOs) features involving in the crucial transitions are presented in Figure 7. One can note that the crucial excitation of K@Ge_2_Au originates from a HOMO-3 → LUMO+2 (42%) transition at ~205 nm, and the two MOs are mainly localized on the two Ge atoms with *s*-lone pairs, and the Au atom with the *d*_xy_- and *p*_y_-lone pairs, respectively. For K@Ge_3_Au, however, the large absorption band at ~217 nm with *f*_0_ = 0.297 ascribes to two mainly electron transitions, listed in Table 1, being the HOMO → LUMO+12 (18%) and HOMO-5 → LUMO+2 (12%) transitions, respectively. Of which the HOMO is mostly delocalized on the clusters surface (rhombic moiety) via the σ chemical bonds (e.g., Ge–Ge, Au–Ge, etc.), whereas the LUMO+12 is intensely localized on the K atom with the *p*_x_-lone pair (see Figure 7). It is noteworthy that the strong transitions can be designated as intramolecular charge transfer, and this should be related to the large *β*_tot_ value. Moreover, the difference of charge transfer can give rise to various electronic effects, but all of other MOs contribute little to electronic transitions of the cluster (≤8%). Table 1 lists the major contributions of electronic transition of the Na@Ge_7_Au cluster in crucial excited states. Its strongest transitions can be assigned to be composed by three mainly mixed excitations of HOMO-7 → LUMO (28%), HOMO-6 → LUMO (17%), and HOMO-2 → LUMO+4 (14%). From Figure 7, it is evident that HOMO-7 is delocalized on QP’s surface via σ bonds, and HOMO-2 and HOMO-6 can be formed by two π bonds, located at the inner and outer QP, whereas the electron distributions of LUMO are mainly associated with the Na–Au bond instead of other atoms, and LUMO+4 is mostly delocalized on the QP’s surface with additional distribution on Au atom. Therefore, the situation of intramolecular charge transfer of cluster directly influences the transition energy, which is a decisive factor that leads to a considerably large first hyperpolarizability.

## 4. Conclusions

In the present work, we have systematically investigated the structures, chemical stabilities and nonlinear optical properties as well as the chemical bonding and electronic structures of a series of alkali metals-adsorbed gold-germanium bimetallic clusters using the hybrid DFT-B3LYP method. Structurally, it has been determined that the adsorption of alkali metal atoms does not largely affect the structural framework of the gold-germanium clusters, and the alkali metals prefer energetically to be attached on clusters’ surface or edge. The Li-adsorbed bicapped pentagonal prism of the Li@Ge_12_Au cluster is electronically stable due to obeying the spherical aromaticity counting rule. Meanwhile, the molecular orbitals analysis reveals that the Au-4*d* state has strong interactions with the germanium atoms, and the hybridization between the Ge and Au atoms can enhance the chemical stability of the bimetallic cluster. This AdNDP analysis indicates that the localized 2c-2e σ-bonds are located on the clusters’ surface, while all the delocalized σ-bonds are involved in the endohedral Au dopant, which are responsible for structural stabilization of Li@Ge_12_Au. The static first hyperpolarizabilities are strongly related to the cluster size and geometric structure, and the AM@Ge*_n_*Au (AM = Na and K) clusters display the large *β*_tot_ values, which are enough to establish their strong nonlinear optical behaviors, especially for Na@Ge*_n_*Au (*n* = 7 and 12) and K@Ge*_n_*Au (*n* = 2 and 3). The present results will inevitably stimulate future experimental and theoretical studies of germanium-based semiconductor clusters doped by alkali metals for the design of novel nonlinear optical materials.

## Figures and Tables

**Table 1:** The isotropic polarizabilities (*α*_iso_, in a.u.), static first hyperpolarizability (*β*_tot_, in a.u.), transition energy (∆*E*, in eV), maximum oscillator strength (*f*_0_, in a.u.), and the change in dipole moment (∆*μ*, in Debye) for crucial excited states of the following clusters.

Clusters	*α*_iso_	*β*_tot_	∆*E*	*f*_0_	∆*μ*	Crucial Transitions * (%)
K@Ge_2_Au	170	9602	6.056	0.358	0.371	H-3 → L+2 (42%)
K@Ge_3_Au	209	8812	5.702	0.297	0.623	H → L+12 (18%), H-5 → L+2 (12%)
Na@Ge_7_Au	338	13050	3.798	0.128	3.245	H-7 → L (28%), H-6 → L (17%), H-2 → L+4 (14%)

* H = HOMO, L = LUMO.

## Supplementary Materials

The following are available online at http://www.mdpi.com/2079-4991/7/7/184/s1. Table S1: Comparison of relative energies for the low-lying neutral AM@Ge*_n_*Au (AM = Li, Na, and K; *n* = 2–13) clusters, calculated by using the hybrid DFT-B3LYP functionals together with two different basis sets.
