# Supplementary material for: Effect of Alkali Metal Atoms Doping on Structural and Nonlinear Optical Properties of the Gold-Germanium Bimetallic Clusters
Source: Nanomaterials (Basel). 2017 Jul 17;7(7):184. doi: 10.3390/nano7070184 (PMC5535250; doi:10.3390/nano7070184)
Supplement: Supplementary file 1 [file nanomaterials-07-00184-s001.pdf]

Supplementary Materials

Effect of Alkali Metal Atoms Doping on Structural and Nonlinear Optical Properties of Gold-Germanium Bimetallic Clusters: Focusing on the Design of Novel Optical Germanium-Based Materials

Xiaojun Li, Shuna Li, Hongjiang Ren, Juxiang Yang, Yongqiang Tang

The Key Laboratory for Surface Engineering and Remanufacturing in Shaanxi Province, School of Chemical Engineering, Xi'an University, Xi'an 710065, Shaanxi, P. R. China

Table S1 Comparison of relative energies (E_{rel}, eV)^{a} for the low-lying neutral AM@Ge$_n$Au (AM = Li, Na, and K; n = 2 − 13) clusters, calculated by using the hybrid DFT-B3LYP functionals.

Clusters	Struct	Spin	E_{rel}^{1}	E_{rel}^{2}	Clusters	Struct	Spin	E_{rel}^{1}	E_{rel}^{2}	Clusters	Struct	Spin	E_{rel}^{1}	E_{rel}^{2}
Li@Ge$_2$Au	2A	1	0.00	0.00	Na@Ge$_2$Au	2A	1	0.00	0.00	K@Ge$_2$Au	2A	1	0.00	0.00
		3	1.12	1.23			3	0.98	1.03			3	1.05	1.43
Li@Ge$_3$Au	3A	1	0.00	0.00	Na@Ge$_3$Au	3A	1	0.00	0.00	K@Ge$_3$Au	3A	1	0.00	0.00
		3	0.63	1.24			3	0.94	1.16			3	0.82	1.01
Li@Ge$_4$Au	4A	1	0.00	0.00	Na@Ge$_4$Au	4A	1	0.00	0.00	K@Ge$_4$Au	4A	1	0.00	0.00
		3	0.35	0.39			3	0.42	0.40			3	0.36	0.33
Li@Ge$_5$Au	5A	1	0.00	0.00	Na@Ge$_5$Au	5A	1	0.00	0.00	K@Ge$_5$Au	5A	1	0.00	0.00
		3	0.91	0.78			3	0.81	0.86			3	0.81	0.88
	5B	1	0.07	0.13		5B	1	0.15	0.16		5B	1	0.13	0.13
		3	0.73	1.03			3	0.98	1.05			3	0.94	1.03
Li@Ge$_6$Au	6A	1	0.00	0.00	Na@Ge$_6$Au	6A	1	0.00	0.00	K@Ge$_6$Au	6A	1	0.00	0.00
		3	0.88	1.03			3	0.86	1.00			3	0.90	1.02
	6B	1	0.16	0.22		6B	1	0.06	0.13		6B	1	0.09	0.15
		3	0.78	0.82			3	0.79	0.82			3	0.82	0.80
Li@Ge$_7$Au	7A	1	0.00	0.00	Na@Ge$_7$Au	7A	1	0.07	0.01	K@Ge$_7$Au	7A	1	0.00	0.00
		3	0.25	0.27			3	0.35	0.30			3	0.29	0.09
	7B	1	0.03	0.12		7B	1	0.00	0.00		7B	1	0.03	0.09
		3	0.84	0.61			3	0.81	0.51			3	0.49	0.53
Li@Ge$_8$Au	8A	1	0.00	0.00	Na@Ge$_8$Au	8A	1	0.04	0.00	K@Ge$_8$Au	8A	1	0.02	0.00
		3	0.73	0.78			3	0.73	0.74			3	0.88	0.70
	8B	1	0.04	0.06		8B	1	0.00	0.02		8B	1	0.00	0.01

		3	0.68	0.61			3	0.66	0.59			3	0.61	0.58
$Li@Ge_9Au$	9A	1	0.00	0.00	$Na@Ge_9Au$	9A	1	0.00	0.00	$K@Ge_9Au$	9A	1	0.00	0.00
		3	0.92	1.01			3	0.79	0.94			3	0.73	0.90
	9B	1	0.04	0.06		9B	1	0.07	0.04		9B	1	0.08	0.06
		3	0.89	1.18			3	0.92	1.17			3	0.88	1.19
$Li@Ge_{10}Au$	10A	1	0.00	0.00	$Na@Ge_{10}Au$	10A	1	0.00	0.00	$K@Ge_{10}Au$	10A	1	0.00	0.00
		3	1.23	0.98			3	1.27	1.22			3	0.41	1.20
$Li@Ge_{11}Au$	11A	1	0.00	0.00	$Na@Ge_{11}Au$	11A	1	0.00	0.00	$K@Ge_{11}Au$	11A	1	0.00	0.00
		3	0.72	0.78			3	0.73	0.84			3	0.81	0.81
	11B	1	0.00	0.07		11B	1	0.03	0.09		11B	1	0.09	0.12
		3	0.77	0.80			3	0.80	0.89			3	0.85	0.85
$Li@Ge_{12}Au$	12A	1	0.00	0.01	$Na@Ge_{12}Au$	12A	1	0.00	0.00	$K@Ge_{12}Au$	12A	1	0.00	0.00
		3	0.67	0.79			3	0.63	0.64			3	0.69	0.78
	12B	1	0.04	0.00		12B	1	0.03	0.01		12B	1	0.08	0.10
		3	0.50	0.57			3	0.48	0.56			3	0.49	0.56
$Li@Ge_{13}Au$	13A	1	0.00	0.00	$Na@Ge_{13}Au$	13A	1	0.04	0.00	$K@Ge_{13}Au$	13A	1	0.00	0.00
		3	0.38	0.46			3	0.41	0.48			3	0.42	0.46
	13B	1	0.18	0.22		13B	1	0.00	0.03		13B	1	0.15	0.15
		3	0.71	0.79			3	0.70	0.81			3	0.56	0.59

[a]The E_{rel}^1 and E_{rel}^2 symbols represent the relative energies with the zero-point vibrational corrections, which were obtained by using two different basis sets (LanL2DZ(Ge,Au)/def-SVP(AM) and def-TZVP for all atoms), respectively.
